# Nautical officers at sea: emergency experience and need for medical training

**DOI:** 10.1186/1745-6673-9-19

**Published:** 2014-05-03

**Authors:** Marcus Oldenburg, Jan Rieger, Christoph Sevenich, Volker Harth

**Affiliations:** 1 Institute for Occupational and Maritime Medicine, ZfAM, University Medical Center Hamburg-Eppendorf, Seewartenstrasse10, Hamburg D-20459, Germany

**Keywords:** Medical emergencies on the high seas, Medical refresher course, Nautical officers, Educational contents

## Abstract

**Background:**

On merchant ships, the medical treatment including emergency interventions on the high seas are carried out by nautical officers who have to pass a forty hours medical refresher course every five years in order to meet international requirements. This study aims to show the most frequent kinds of medical emergencies on the high seas and to assess the seafarers’ knowledge about their treatment.

**Methods:**

465 nautical officers who participated in the medical refresher course at the Institute for Occupational and Maritime Medicine in Hamburg, within the period from 2006 to 2013, were interviewed about their experience of serious diseases and accidents on board, which had led to an emergency port call, a course deviation or an evacuation. Furthermore, prior to the course the officers were asked to answer 18 basic medical questions about common medical issues on board.

**Results:**

133 seafarers (28.6%) reported that they had been confronted with at least one serious medical emergency at sea. These emergencies encompassed trauma (37.9%), cardiovascular diseases (18.2%), severe gastrointestinal diseases (15.9%), serious skin or pulmonary infections (9.8%), neurological (9.1%) and urological diseases (4.5%) as well as burns (4.5%). With regards to the basic medical questions, an average of 70.7% of the total score had been achieved (from 26.8% to 100%). On average, 65.5% of internal and 65.6% of surgical questions had been answered correctly. Proper answers to toxicological and infectious questions had been given by 93.3% and 94.1% respectively and to topics of hypothermia and medical treatment by 59.4% and 61.0%. In total, a significant number of younger seafarers answered the questions correctly (p = 0.001).

**Conclusions:**

According to this study, serious emergencies on board are most frequently related to trauma or cardiovascular diseases. Taking into account the acquired medical knowledge, there seems to be a need to train deck officers within these fields more intensively. Considering the knowledge of seafarers about medical issues directly before attending their medical refresher course, the 5 years interval without any form of refresher course appears to be too long to guarantee adequate medical treatment by the lay persons on board.

## Background

Seafaring poses a high risk-occupation within an isolated environment [1]. According to recent telemedical reports, the most frequently observed medical emergencies were related to surgical (46%), internal (27%) and urological (6%) health disorders [2,3]. While on passenger and larger research ships a ship’s doctor is mandatory and responsible, on merchant vessels the medical treatment of these diseases, including emergency interventions, is carried out by nautical officers, the captain and his substitutes who are, in fact, medical laymen. For this reason, all nautical officers on seagoing vessels receive advanced medical training as part of their studies at maritime universities/academies. The main skills that have to be achieved by the officers are written down in international requirements. The most important international requirement for training standards in general are the Standards of Training, Certification and Watchkeeping (STCW) released by the International Maritime Organization (IMO) [4]. Any seafarer, including ratings, who wants to join a merchant vessel, must prove that he meets the minimum standards of competency in elementary first aid to take immediate action upon encountering an accident or other medical emergency.

In addition, officers have to be trained in medical care and the treatment of injured persons while they remain on board. The medical scope ranges from applying bandages, placing an intravenous access, suturing wounds and administering strong painkillers [4].

Within the framework of the initial medical education at university which consists of over 120 hours of tuition, the future officers learn about anatomy, frequently occurring diseases, tropical diseases, general patient care, pharmacological basics, reanimation, hypothermia and the ability to take a complete medical history for telemedical advice [5]. The medical training also includes a practical section of 2 weeks in a hospital’s emergency room and bedside teaching [5]. Already in 1992 a European Directive came into force that demands the regular attendance of nautical officers in medical refresher courses every five years to enable them to maintain and increase their knowledge and skills and to keep up-to date with new developments in medical treatment [6]. Since the Maritime Labour Convention (MLC [7]) entered into force at 20^th^ August 2013, this rule is obligatory for all ships worldwide. These requirements are also implemented in the German decree of health care on merchant ships (Krankenfürsorgeverordnung 2007) which directs that ship owners have to ensure that any captain or ship’s officer responsible for medical care should have a valid “Certificate of Medical Care” not older than five years. The decree of health care is going to be replaced by a new “Maritime Medicine Regulation” (MariMedV) which is currently passing an approval process by the German parliament. Within this new regulation, the five year interval and the fourty hours duration for medical refresher courses will remain unchanged. This raises the question whether this period is appropriate to ensure that the officers responsible for health care are able to manage medical emergencies at sea sufficiently. Therefore, this study aims to evaluate the knowledge of seafarers about medical issues, directly before attending their medical refresher course as regulated by law.

## Methods

### Study group

Within the period from 2006 to 2013, the Institute for Occupational and Maritime Medicine in Hamburg performed 41 medical refresher courses with 465 nautical officers. These officers were asked to participate in the present voluntary study. It was guaranteed that the seafarers’ results in this anonymous survey would not have any influence on the issue of their certificate of Medical Care. All the nautical officers asked agreed to participate.

In Germany, there are 10 medical refresher courses approved by the department of health responsible for each federal state. As nautical officers on vacation usually choose a training institute close to their place of residence it is assumed that the participants of the Hamburg Medical Refresher Course are representative of nautical officers in Germany.

Although it cannot be excluded that some participants had changed their vessel type or their voyage route in the course of time, in the authors’ experience it is likely that the vast majority of the officers examined retained their accustomed working place and area of trade during their occupational career.

### Questionnaire

Directly before attending the Hamburg medical refresher course, the seafarers were asked to fill in a questionnaire regarding medical topics. In its first part, the officers were interviewed about their experience of serious diseases on board; these were defined as acute health disorders or accidents which had led to an emergency port call or change of the planned route; for example to transfer an ill subject by helicopter. In the second part of the questionnaire the officers were requested to answer 18 basic medical questions that had been developed by 3 physicians with long-time experience in the field of maritime medicine. The issues reflect topics of the minimum standards of seafarers’ (medical) education given within the international Standards of Training, Certification and Watchkeeping (STCW). They were related to the main disciplines of internal (n = 5) and surgical (n = 5) medicine as well as 2 questions to relevant to the maritime topics of toxicology (due to transport of toxic substance), infectious diseases (especially during ships’ routes in the tropical zones), hypothermia (for example caused by immersion in cold water) and medical treatment that poses a strong challenge for the medical lay person, such as thorax compression or bladder puncture. Based on telemedical experiences at sea, in this study most questions referred to surgical and internal topics, as these were the majority of emergencies and experienced diseases on board ships [3].

Finally, the nautical officers were asked to state how well prepared they felt to cope with a medical emergency using school marks from 1–5, where 1 is the best and 5 is the worst grade.

### Statistical analysis

Data analysis was performed with SPSS for Windows (version 18.0, SPSS GmbH Software, Munich, Germany). Continuous variables were presented as mean (±standard deviation (SD)). The Pearson Chi-square test was applied to compare frequencies between groups. For evaluation of differences between groups, the Kruskal-Wallis test was used. All indicated p-values were two-sided, and an α-value of < 0.05 was regarded as statistically significant.

## Results

### Study group

The study population consisted of 453 males and 12 females with an average age of 46.2 years (SD ± 11.9 years). According to the rank, in the study sample were 257 captains, 192 nautical officers, 11 technical officer and 5 others (e.g. ship inspectors). As expected, the captains were considerably older than the nautical and technical officers (Table 1).

**Table 1:** Demographic, educational and occupational data in relation to the seafarers’ rank

	***Captain (n = 257)***	***Nautical officer (n = 192)***	***Technical officer (n = 11)***	***Others (n = 5)***	***p***
**Sex**					0.069^#^
*Males (453); n (%)*	255 (99.2%)	183 (95.3%)	10 (90.9%)	5 (100%)	
*Females (12); n (%)*	2 (0.8%)	9 (4.7%)	1 (9.1%)	0	
**Age,** mean (±sD)	51.7 (10.1)	38.9 (10.3)	38.0 (8.9)	49.6 (8.9)	**< 0.001**^**&**^
**Time duration (years) before previous**					
Refresher course, mean (±sD)	5.6 (1.8)	5.3 (0.9)	5.3 (0.5)	5.0 (0)	0.483^&^
**Previous trading area;***n (%)*					**0.008**^**#**^
*Costal (106 )*	65 (25.3%)	39 (20.3%)	0	2 (40%)	
*Medium (42)*	31 (12.1%)	10 (5.2%)	0	1 (20%)	
*Worldwide (317)*	161 (62.6%)	143 (74.5%)	11 (100%)	2 (40%)	
**Previous type of the vessel;***n (%)*					**0.004**^**#**^
*Container ship (255)*	131 (51.0%)	112 (58.2%)	10 (90.9%)	2 (40%)	
*General Cargo ship (38)*	29 (11.3%)	9 (4.7%)	0	0	
*Tanker (21)*	16 (6.2%)	5 (2.6%)	0	0	
*Passenger liner (34)*	17 (6.6%)	16 (8.3%)	1 (9.1%)	0	
*Supply boat (5)*	0	5 (2.6%)	0	0	
Other vessels (111)*	64 (24.9%)	45 (28.6%)	0	3 (60%)	

*including 10 research vessel/^#^Chi-square test/^&^Kruskal-Wallis test.

Significant results are bold.

At the beginning of the Hamburg medical course, the previous refresher course was on average 5.5 years ago (SD ± 1.5 years).

Prior to the Hamburg course, most participants (68.2%) were sailing worldwide, 22.8% in medium and 9% in coastal trading areas.

### Serious emergencies at sea

**Figure 1:**
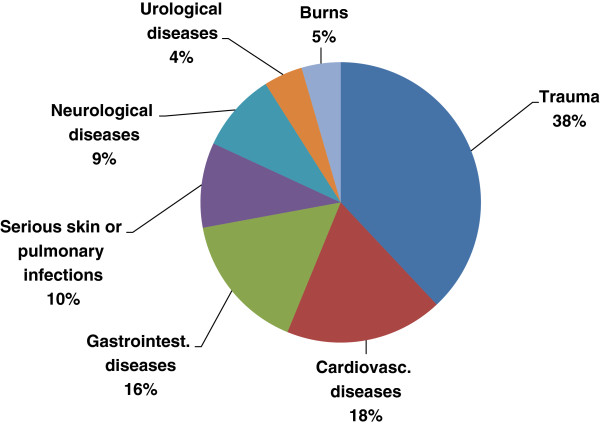
Frequencies (%) of serious medical emergencies in all types of vessels.

A total of 133 seafarers (28.6%) reported that they had previously been confronted with at least one serious medical emergency - with an average number of 1.6 (from 1 to 8). The lay answers of the seafarers have been recognized within the following sub-categories, in decreasing order: trauma, cardiovascular diseases, severe gastrointestinal diseases, serious skin or pulmonary infections, neurological, and urological diseases as well as burns (Figure 1). Summarizing cardiovascular diseases, severe gastrointestinal diseases and urological diseases as internal diseases (38%) and trauma as surgical diseases (38%) shows that the main part of serious medical emergencies at sea can be found within these two fields.

A high number of serious emergencies occurred particularly on passenger liners (67.4%) with many people on board. Besides passenger ships, these emergencies were reported on research vessels (50.0%), tankers, container ships, cargo ships and supply boats in decreasing order (p = 0.029) (Table 2). Only officers on passenger liners (6 times), research vessels (5 times) and container ships (4 times) had experienced more than 3 serious emergencies.

**Table 2:** Occurrence of serious medical emergencies in respect of the vessel type

	***Container ship (255)***	***General Cargo ship (38)***	***Tanker (21)***	***Passenger liner (34)***	***Other boats (111)***
***All emergencies,*** *n (%)*	69 (27.1%)	8 (20.5%)	6 (28.6%)	23 (67.4%)	27 (24.3%)
***Specific emergencies,****n (%)*					
*Trauma*	24 (9.4%)	5 (12.8%)	3 (14.3%)	7 (20.6%)	11 (9.9%)
*Cardiovasc. diseases*	10 (3.9%)	1 (2.6%)	1 (4.8%)	10 (29.4%)	2 (1.8%)
*Gastrointest. diseases*	11 (4.3%)	1 (2.6%)	1 (4.8%)	3 (8.8%)	5 (4.5%)
*Serious skin or pulmonary infections*	10 (3.9%)	0	0	0	2 (1.8%)
*Neurological diseases*	7 (2.7%)	0	0	3 (8.8%)	4 (3.6%)
*Urological diseases*	3 (1.2%)	0	1 (4.8%)	0	2 (1.8%)
*Burns*	4 (1.6%)	1 (2.6%)	0	0	1 (0.9%)

### Seafarers’ knowledge about medical issues

**Figure 2:**
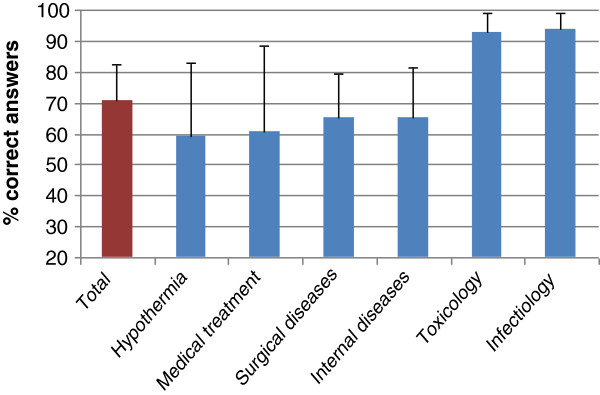
Percentage of correct answers to different medical issues.

With reference to the basic medical questions, an average of 70.7% of the total score had been achieved (from 26.8% to 100%). More than 90% of the seafarers gave correct answers to toxicological and infectious questions, whereas internal and surgical questions as well as those about hypothermia and medical treatment were answered correctly by 60% to 65% of the participants (Figure 2).

After applying the median of age (47 years), it was found that the younger seafarers achieved significantly more correct answers than the older ones (72% vs. 68%; p = 0.001). This was particularly true for internal diseases and hypothermia (each p < 0.001). No differences in the knowledge - assessed by the number of correct answers - were found between captains and nautical officers - with the exception of significantly more experienced nautical officers in hypothermia (64% vs. 55%; p < 0.001). Correct answers to questions concerning medical treatment were more often given by older participants and captains.

The group of officers sailing on medium and large voyage routes gave more often correct answers on infectious and internal diseases than seafarers on coastal voyages (96% vs. 87%; p = 0.001 respective 67% vs. 62%; p = 0.028).

As most examined seafarers met exactly the five 5 year interval since their past refresher course, no association between time duration from the previous course and the number of correct answers could be determined. In addition, correct answers did not depend on the seafarers’ gender or their current vessel type.

On average, the nautical officers feel themselves adequately prepared to deal with medical emergencies by 2.87 (SD ± 0.71) within a range of school marks from 1–5. The self-assessed experience in coping with emergencies was independent of the seafarers’ gender, rank, current vessel type and voyage route and also did not correlate with the number of correct answers in the applied questionnaire.

## Discussion

This study is based on data for quality management measures of the Institute for Occupational and Maritime Medicine in Hamburg (ZfAM). It can be assumed that the pre-course knowledge level among the examined nautical officers is representative of the pre-course knowledge of participants attending refresher courses at other training centres in Germany. On the international level, however, it can't be excluded that the results might diverge.

In this study, only 34 seafarers (7.3%) worked on passenger ships with a doctor on board available. The vast majority of seafarers (431 seafarers, 92.7%) was employed on merchant ships without direct professional help on board. Consequently, diagnosis and medical treatment of their crews at sea - irrespective of possibilities for telemedical advice from ashore - mainly depended on their knowledge and qualification.

The data in Table 2 shows that almost every 4^th^ officer working on container ships or tankers had been confronted with a severe medical emergency that result in course deviation for medical treatment; in general cargo ships only every 5^th^ officer. Thus, the chance to deal with medical emergencies which require more than only basic first-aid knowledge is high.

Course deviations of passenger liners have been reported by 23 officers (67.4% of those seafarers employed on this vessel type). Within the anonymous design of the questionnaire, it is difficult to ascertain whether some of the officers had been working on the same ship, describing the same cases. According to international studies on emergency medicine on cruise ships however, a chance of almost 70% in becoming involved in a medical emergency that leads to a cruise ship deviation represents a reality [8,9]. This high prevalence of serious emergencies can be explained by the huge number of subjects on board with a high proportion of older passengers, as well as passengers with chronic renal diseases under dialysis treatment or other severe chronic diseases.

In the light of the advanced medicine and the professional medical personnel available on passenger liners, the cost-intensive decision for ship deviation due to ill subjects on board must be thoroughly explained to the shipping company, who might question the need for a deviation because of the high standard of medical care available on passenger ships. As physicians on cruise ships are often medical specialists ashore (e.g. internists, general practitioners, surgeons) who temporarily work on board, they should have special training, particularly because chances of rescue at sea are limited.

The research vessels are also manned with crew members above the average number of crew members. If there are more than 100 persons on board, a ship doctor is obligatory so that on both vessel types usually at least one ship’s doctor is engaged who is in charge of medical care. Due to often hazardous and isolated destinations (e.g. polar region) or operations (deep-sea diving and handling of heavy measurement instruments) on research vessels, a higher prevalence of traumata can be explained. As expected, serious emergencies were particularly observed on vessels bigger than 10,000 gross tonnages and more often on ships sailing worldwide (data not shown).

While on passenger liners usually specialized ship doctors are present, on board of merchant vessels nautical officers have to perform the medical treatment themselves. Therefore, the primary target audience of medical refresher courses are nautical officers as medical lay-persons on board of merchant vessels. The present as well as recent studies reveal that the predominant medical emergencies were trauma [8,10], most probably as a result of the ship’s movements and of the dangerous working environment, especially on container and general cargo ships, due to handling heavy, partly health-endangering cargo [1]. In the authors’ experience, on the subgroup of container ships, illnesses related to general medicine such as diarrhoea, arterial hypertension, and minor lacerations also occur rather frequently at sea.

For the assessment of the seafarers’ knowledge of medical issues, questions of similar level of difficulty were set. By means of the 18 basic questions used about frequent and relevant medical issues on board, the seafarers’ knowledge of medical topics can only be cursorily assessed. Taking into consideration, however, that the longer the questionnaire the more time for training would be lost and the willingness to take part would diminish accordingly, it was not possible to include more questions in this survey. Due to the broad scope of medical topics in the questionnaire, issue-related differences in the participants’ knowledge could be determined [11].

The present analysis shows that questions regarding toxicology and infectious diseases were most often answered correctly. This may be due to the fact that the treatment of persons who have contact with toxic and infectious substances forms a great part of the handling of dangerous goods and ship sanitation regulations. Thus, during other compulsory nautical courses, the officers are specially trained within these fields, which may lead to the observed greater knowledge of these topics.

The fact that 70.7% of the participants responded correctly on the basic theoretical questions corresponds with their satisfactory self-assessment about their ability to handle medical emergencies. This self-assessment mainly depends on their practical skills. Within the refresher course in Hamburg, nearly 50% of the tuition is used for practical training [12]. It is assumed that the practical exercises considerably promote the seafarers’ self-confidence in handling real emergencies [13]. It is also recommended that medical training is performed directly on board (e.g. by special educated ship inspectors, or computer-based training [14]) to keep medical knowledge up to date all times.

## Conclusions

According to this study, serious emergencies on board (leading to deviation to an emergency port call) are most frequently related to trauma or cardiovascular diseases. Taking into account the international minimum standards set up by the Standards of Training Certification and Watchkeeping and the acquired medical knowledge, there seems to be a need to train deck officers within these fields more intensively. Considering the knowledge of seafarers about medical issues directly before attending their medical refresher course, the 5 years interval without any form of refresher course appears to be too long to guarantee adequate medical treatment by the lay persons on board. In Germany, low-qualified first-aid providers - that are legally required in each company ashore - have to attend an initial first aid course of 16 hours duration and a first aid refresher course at least every two years [15]. In comparison with these first-aid providers to seafarers, a two year interval for updating the seafarers’ medical knowledge appears also to be appropriate.

## Competing interests

The authors declare that they have no competing interests.

## Authors’ contributions

MO conceived and designed the study, collected, analysed and interpreted the data, and also drafted the manuscript. JR made substantial contributions to collection and interpretation of data. MO and JR contributed equally. CS and VH contributed in analysing the data. All authors have read and approved the final manuscript.
